# Efficacy of Rezafungin in Prophylactic Mouse Models of Invasive Candidiasis, Aspergillosis, and *Pneumocystis* Pneumonia

**DOI:** 10.1128/AAC.01992-20

**Published:** 2021-02-17

**Authors:** Lynn Miesel, Melanie T. Cushion, Alan Ashbaugh, Santiago R. Lopez, Voon Ong

**Affiliations:** a Pharmacology Discovery Services, Taipei, Taiwan; b University of Cincinnati College of Medicine, Cincinnati, Ohio, USA; c Cincinnati VAMC, Cincinnati, Ohio, USA; d TransPharm Preclinical Solutions, Inc., Jackson, Michigan, USA; e Cidara Therapeutics, Inc., San Diego, California, USA

**Keywords:** *Aspergillus*, *Candida*, *Pneumocystis*, antifungal agents, antifungal therapy, echinocandin, prophylaxis

## Abstract

Antifungal prophylaxis is recommended to prevent invasive fungal disease caused by *Candida* spp., *Aspergillus* spp., and Pneumocystis jirovecii in patients at risk for opportunistic infections, such as allogeneic blood or marrow transplant recipients, patients with hematological disease undergoing chemotherapy, or patients on immunosuppressive therapies. Current approaches to antifungal prophylaxis require multiple agents to cover these key fungi. Rezafungin, a novel echinocandin designed for next-generation properties (e.g., greater stability and long-acting pharmacokinetics for once-weekly dosing), has demonstrated *in vitro* activity against *Candida* and *Aspergillus* spp. and efficacy against *Pneumocystis* spp. biofilms. Rezafungin was evaluated in *in vivo* studies of prophylactic efficacy using immunosuppressed mouse models of invasive candidiasis, aspergillosis, and *Pneumocystis* pneumonia. Rezafungin reduction of *Candida* CFU burden was generally greater with increasing drug concentrations (5, 10, or 20 mg/kg) and when rezafungin was administered closer to the time of fungal challenge (day −1, −3, or −5). Similarly, in the aspergillosis model, survival rates increased with drug concentrations and when rezafungin was administered closer to the time of fungal challenge. Against *Pneumocystis* murina, rezafungin significantly reduced trophic nuclei and asci counts at all doses tested. Rezafungin prevented infection at the two higher doses compared to vehicle and had comparable activity to the active control trimethoprim-sulfamethoxazole at human equivalent doses for prevention. These findings support phase 3 development of rezafungin and the potential for single-agent prophylaxis against invasive fungal disease caused by *Candida* spp., *Aspergillus* spp., and Pneumocystis jirovecii.

## Abstract (precis)

Antifungal prophylaxis is recommended to prevent invasive fungal disease caused by *Candida* spp., *Aspergillus* spp., and Pneumocystis jirovecii in patients at risk for opportunistic infections, such as allogeneic blood or marrow transplant recipients, patients with hematological disease undergoing chemotherapy, or patients on immunosuppressive therapies. Current approaches to antifungal prophylaxis require multiple agents to cover these key fungi.

## INTRODUCTION

Antifungal prophylaxis is an important strategy against invasive fungal disease (IFD) in patients at risk for opportunistic infections, such as recipients of allogeneic blood or marrow transplantation or solid organ transplantation, as well as patients with hematological disorders undergoing chemotherapy (1–6). For such patient populations, antifungal prophylaxis is recommended to prevent infections caused by *Candida* spp., *Aspergillus* spp., and Pneumocystis jirovecii (7–9). An ideal antifungal prophylaxis regimen would provide fitting coverage of the most prevalent opportunistic pathogens without obstructing or complicating therapy due to toxicity, intolerability, or drug-drug interactions (DDIs).

While recommendations and clinical trial data are available to guide antifungal prophylaxis (7, 10–14), there is no single approach as prophylaxis must be customized to the needs of a given patient, as well as local fungal epidemiology and susceptibility. Current strategies to protect against these most commonly encountered pathogens generally enlist an azole (one with mold activity if there is risk of *Aspergillus*) and trimethoprim-sulfamethoxazole (SXT) for Pneumocystis jirovecii pneumonia (PCP) (7, 9, 10). Considerations when personalizing azole therapy include pharmacokinetic variability, oral tolerability, and safety (such as liver toxicity and effects on the QT interval), as well as DDIs (15, 16). For SXT, personalizing therapy may include modifications to antifungal prophylaxis and/or primary treatment to mitigate SXT-associated fever, rash resembling GVHD, nephrotoxicity, or myelosuppression (8). Unmet needs in the current approach to antifungal prophylaxis may lead to gaps in protection against IFD or complications in treatment of primary disease, such as when dosing is disrupted or when adverse effects (AEs) and DDIs occur.

With the advent of newer therapies for treatment of primary disease, many of which introduce new or increased risks of IFD and DDIs with antifungal agents, patient-level considerations have expanded in scope and complexity. Recent examples include the increased incidence of PCP observed with Bruton’s tyrosine kinase inhibitors (ibrutinib and acalabrutinib) and IFD associated with immune checkpoint inhibitors (17–21). Arguably, the greatest impact of newer treatments on antifungal prophylaxis may be from DDIs caused by CYP interactions or other mechanisms that may contraindicate concomitant use (16, 22). The AEs of newer treatments or their management with corticosteroids or other immunosuppressants may also increase infection risk (23–26). Additional experience with newer treatments will help to guide antifungal prophylaxis management. At the same time, newer antifungal options and strategies are needed to support the continuous advancements in treatment of hematologic diseases and in immunosuppressive therapies.

Rezafungin is a novel echinocandin in development for the treatment and prevention of invasive candidiasis, with a phase 3 treatment trial (ReSTORE NCT03667690) and a phase 3 prophylaxis trial (ReSPECT NCT04368559) under way. While the current approach to antifungal prophylaxis requires multiple agents to cover the key target pathogens, rezafungin has demonstrated *in vitro* activity against *Candida* and *Aspergillus* species, including azole-resistant strains of Aspergillus fumigatus, as well as efficacy against *Pneumocystis* biofilms (27–34). Rezafungin is distinguished by a long half-life and front-loaded, high plasma drug exposures that allow for the once-weekly intravenous dosing regimen in clinical development. Rezafungin administered once weekly has demonstrated safety and tolerability consistent with that of its echinocandin class. Preclinical evaluation demonstrated rezafungin chemical and metabolic stability and lack of hepatotoxicity, in contrast to anidulafungin (35, 36). Phase 1 trials of rezafungin showed a lack of effect on the QT interval and low risk of DDIs with commonly used drugs (37, 38). To further contribute to these data, this series of *in vivo* studies evaluated the efficacy of rezafungin in prophylactic mouse models of invasive fungal infections caused by *Candida*, *Aspergillus*, and *Pneumocystis* in immunosuppressed mice.

(Data from these studies were preliminarily presented at the 2017 European Hematology Association meeting [Madrid, Spain].)

## RESULTS

### Prevention of *Candida* infection.

**FIG 1:**
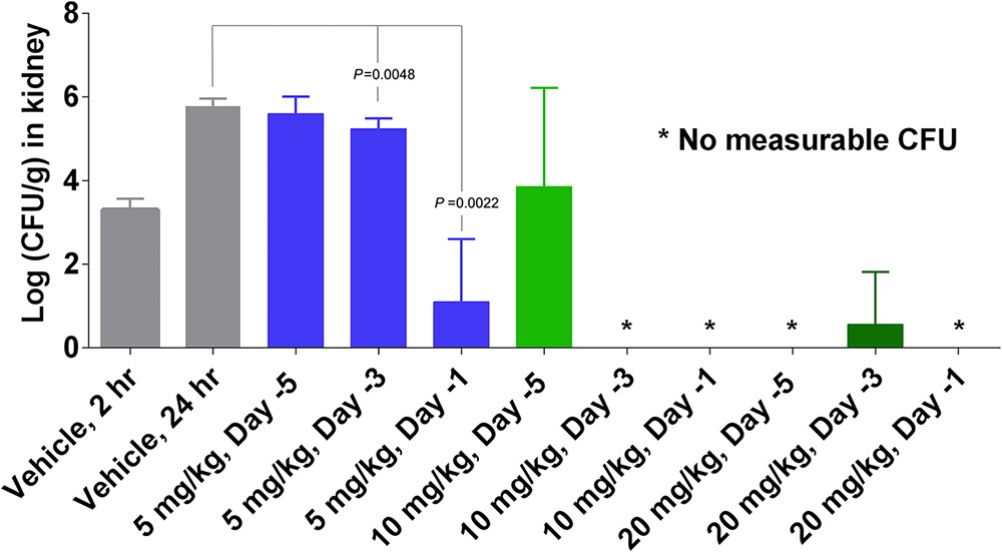
Clearance and significant decreases in kidney CFU burden of *Candida* (C. albicans, MIC = 0.03 µg/ml) in mice after administration of rezafungin prophylaxis.

Rezafungin prophylaxis in an immunosuppressed mouse model of invasive candidiasis demonstrated decreases in the *Candida* CFU burden, an effect that increased with rezafungin concentrations and when prophylaxis administration occurred closer to challenge (day −1 > day −3 > day −5; Fig. 1). C. albicans was completely cleared in all animals given rezafungin 20 mg/kg, except for one animal that had prophylaxis administered on day −3. At the lower doses (10 and 5 mg/kg), bioburden reduction when prophylaxis was administered on day −5 (similar to day −15 for humans as described in Materials and Methods) was not significantly different than vehicle. However, when prophylaxis administration occurred closer to challenge, on day −3 or day −1, the 10-mg/kg rezafungin groups had no measurable CFU, and the 5-mg/kg groups showed significantly lower fungal burden than did the vehicle control, indicating a dose response as well as correlation with timing of prophylaxis administration (day −1 > day −3 > day −5).

### Prevention of *Aspergillus* infection.

**FIG 2:**
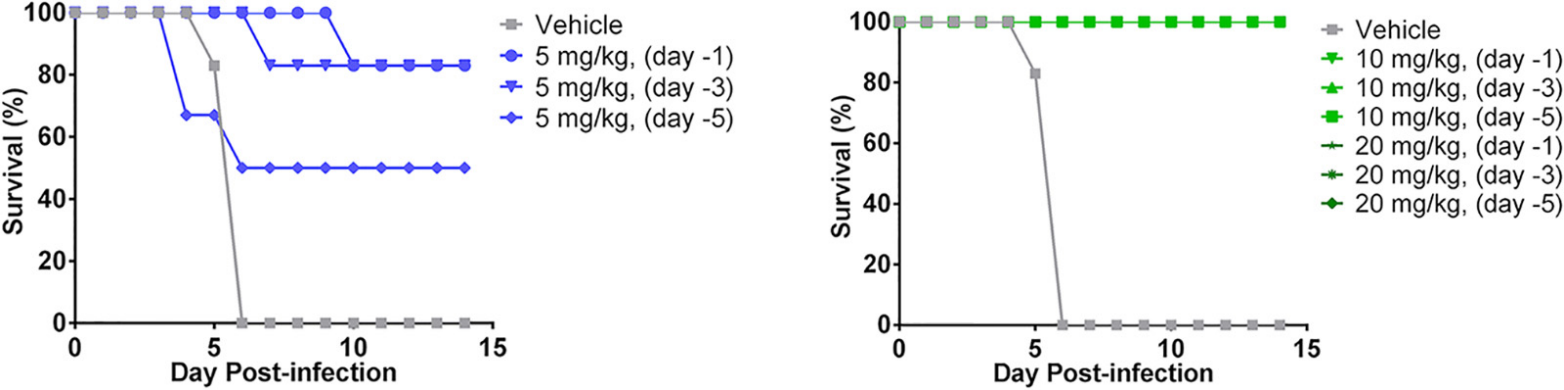
Survival rates in mice challenged with *Aspergillus* (A. fumigatus, MEC = 0.0078 µg/ml) after administration of rezafungin prophylaxis. (Left) 5 mg/kg; (right) 10 or 20 mg/kg. *P* < 0.05 for all dosing arms, except for 5 mg/kg, day –5 (*P* = 0.182).

Rezafungin prophylaxis against A. fumigatus in the immunosuppressed mouse model of invasive aspergillosis demonstrated protection at doses of 10 and 20 mg/kg, with all animals in these groups surviving the 14-day postchallenge period regardless of when prophylaxis was administered (Fig. 2, right). In the group given the lowest dose tested (rezafungin 5 mg/kg), survival increased when prophylaxis administration occurred closer to challenge (day −1 > day −3 > day −5; Fig. 2, left).

### Prevention of PCP.

**FIG 3:**
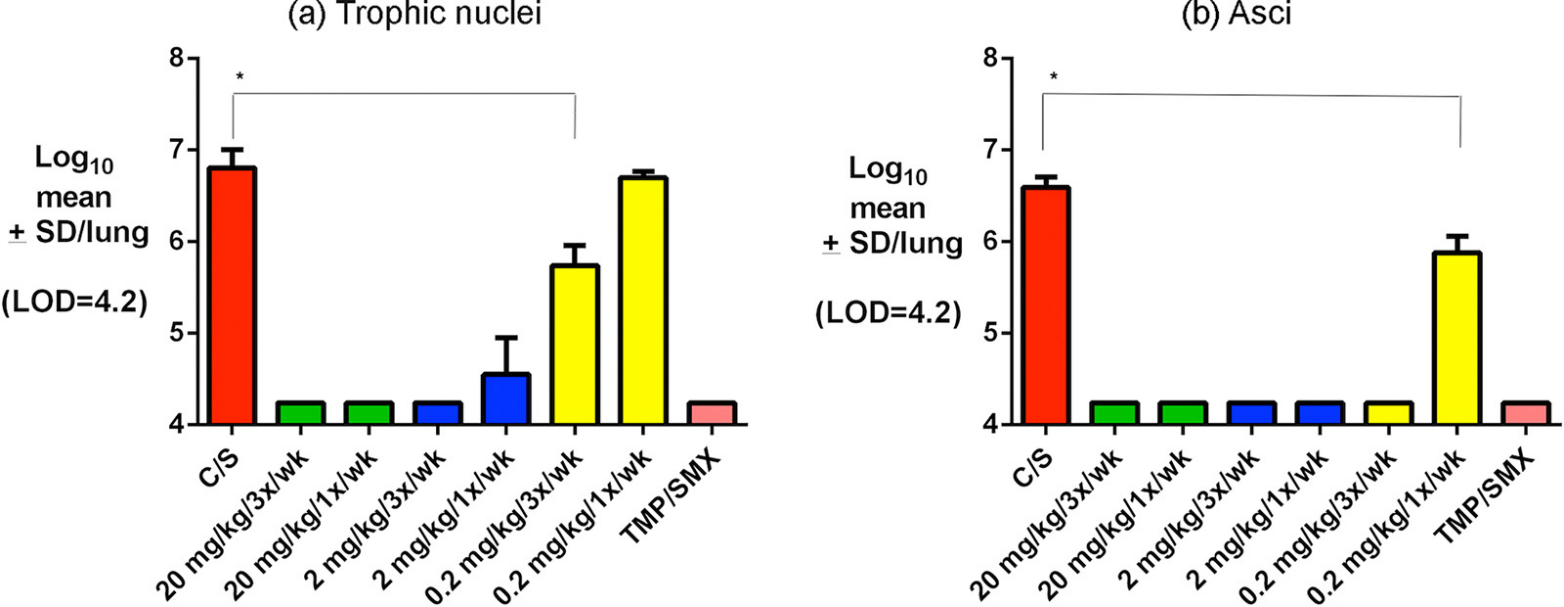
Clearance and significant decreases in kidney burden of *Pneumocystis* (P. murina; both trophic [a] and asci [b] forms) in mice after administration of rezafungin prophylaxis compared to active control SXT. *, *P* < 0.05 versus the control. The limit of microscopic observation on this scale is log_10_ of 4 (the value indicating that no nuclei or asci were observed).

Rezafungin prophylaxis in the immunosuppressed mouse model of PCP demonstrated significantly reduced trophic nuclei counts in all rezafungin-treated groups compared to the vehicle control, except at the lowest and least frequently administered dose (0.2 mg/kg 1×/week). Three of the rezafungin groups—both 20-mg/kg regimens (20 mg/kg 1× or 3×/week) and the 2-mg/kg regimen (3×/week)—were comparable to the active control SXT, with no trophic nuclei microscopically detected (Fig. 3a). Similarly, asci counts in all rezafungin-treated groups were significantly reduced compared to the vehicle control. The efficacy observed in all but the 0.2-mg/kg 1×/week dose group was comparable to that of SXT, with no asci microscopically detected (Fig. 3b).

## DISCUSSION

In this series of *in vivo* experiments, the novel echinocandin rezafungin was efficacious in preventing infection caused by *Candida*, *Aspergillus*, and *Pneumocystis* in immunosuppressed mice. Although the interpretation of these findings is limited to the extent that preclinical research may translate to clinical experience, the clinical efficacy demonstrated by currently available, once-daily echinocandins against all three of the fungal pathogens studied supports the predictive value of these *in vivo* data. The role of preclinical models notwithstanding, these results contribute to the development of rezafungin and the future of antifungal prophylaxis for patients at risk for IFD. To our knowledge, rezafungin is the first echinocandin to report prophylactic efficacy against *Pneumocystis*, as well as against *Candida* and *Aspergillus*.

Safe and efficacious antifungal prophylaxis is important and yet increasingly challenging to provide in a growing number of immunosuppressed conditions and patient populations at risk of developing IFD. The current mainstays of prophylaxis against these key pathogens (azoles and SXT) are generally efficacious but limited by DDIs and/or toxicity that can impede prophylaxis, as well as the treatment of primary disease (39, 40). Alternative therapies for prophylaxis are lacking. Pentamidine, dapsone, and atovaquone, as options for PCP prophylaxis, each have tolerability issues, limitations in efficacy, or administration challenges in the case of inhaled pentamidine (41). Liposomal amphotericin B at intermittent or low doses has been used for prophylaxis of *Candida* and *Aspergillus* but is hampered by a side-effect profile that includes nephrotoxicity and electrolyte abnormalities. In contrast, the relative tolerability and lack of DDIs with echinocandins present an attractive safety profile (41, 42). While all three echinocandins have been studied in various patient populations (13, 43–48), only micafungin is indicated for use as antifungal prophylaxis, specifically, of *Candida* infections in adult and pediatric patients undergoing hematopoietic stem cell transplantation (12, 49, 50). In a retrospective study in patients with hematological disease who received antifungal prophylaxis, micafungin was selected in 26% of 104 cases for safety- or tolerability-related reasons (e.g., liver dysfunctions, severe mucositis, DDIs of other antifungals, and long QT syndrome) (51). The safety of rezafungin is consistent with that of the echinocandin class, as observed in the phase 2 STRIVE trial of once-weekly rezafungin compared to caspofungin in the treatment of candidemia and invasive candidiasis (35, 52) and in phase 1 trials that confirmed rezafungin lack of effect on the QT interval and low DDI potential (37, 38).

The efficacy findings reported here also underscore the distinctive pharmacokinetics of rezafungin. Its long half-life and front-loaded drug exposure allow for once-weekly dosing of rezafungin, as studied in the completed phase 2 and ongoing phase 3 clinical trials. Furthermore, rezafungin demonstrates extensive distribution and tissue penetration, as shown by Zhao et al. (53), who observed 4-fold-higher rezafungin concentrations within lesions than for micafungin at the same dosage in a mouse intra-abdominal abscess model. Rezafungin is distributed to lung epithelial lining fluid and has demonstrated high *in vivo* exposures in the lung and other organs commonly infected by IFD, ∼4-fold higher than in plasma (54–56). These pharmacokinetics, together with the *in vivo* efficacy of rezafungin demonstrated here, suggest a potential to replace poorly tolerated combination regimens for prophylaxis in patients at risk for IFD, many of whom are burdened by polypharmacy and increasingly longer periods of infection risk.

The current set of *in vivo* studies on rezafungin contribute important findings to the published literature on this novel echinocandin and on approaches to antifungal prophylaxis. The efficacy of rezafungin in preventing infections caused by *Candida*, *Aspergillus*, and *Pneumocystis* in immunosuppressed mice demonstrate the potential of rezafungin as prophylaxis for patients at high risk of infection and support its ongoing clinical development.

## MATERIALS AND METHODS

These studies were performed in accordance with the *Guide for the Care and Use of Laboratory Animals*, 8th ed. (National Academies Press, Washington, DC), in AAALAC-accredited ABSL-2 laboratories (with the exception of P. murina-infected mice) under the supervision of veterinarians. In addition, all procedures were conducted in compliance with the Institutional Animal Care and Use Committee at the respective sites.

Test agents were supplied by Cidara Therapeutics, Inc. (San Diego, CA), except for SXT (Sulfatrim H pediatric oral suspension; Actavis, Baltimore, MD), and amphotericin B (Sigma), which were purchased.

### Invasive candidiasis prophylaxis mouse model.

Female ICR mice (Envigo Laboratories) weighing ∼0.02 kg were immunosuppressed using two intraperitoneal (i.p.) injections of cyclophosphamide, with a first injection of 150 mg/kg administered 4 days before challenge (day −4) with Candida albicans (American Type Culture Collection [ATCC] SC5314; Manassas, VA; 4.5 log_10_ CFU/mouse intravenous [i.v.]) and a second injection of 100 mg/kg administered 1 day before challenge (day −1). Prior to C. albicans challenge, mice (*n* = 5/group; 9 groups) were treated with one subcutaneous (s.c.) dose of either rezafungin 5, 10, or 20 mg/kg on either day −5 (which is similar to day −15 for humans, based on a 2- to 3-fold faster clearance in mice), day −3, or day −1. In three additional groups, mice were given either rezafungin at 5 mg/kg s.c., micafungin at 5 mg/kg i.p., or s.c. vehicle control on day 0 administered immediately following C. albicans challenge. Treated mice were sacrificed at 24 h postchallenge, and the kidneys were harvested for bioburden enumeration (CFU/g of tissue).

### Invasive aspergillosis prophylaxis mouse model.

Female ICR mice (BioLasco Taiwan/Charles River) weighing ∼0.02 kg were immunosuppressed using three i.p. injections of cyclophosphamide, with the first injection (6 mg/mouse) administered 3 days before challenge (day –3) with Aspergillus fumigatus (ATCC 13073; Rockville, MD; 1.85 × 10^4^ CFU/mouse i.v.) and the second and third injections (2 mg/mouse) administered 1 day before (day –1) and 4 days after (day 4) challenge. Prior to A. fumigatus challenge, mice (*n* = 6/group; 9 groups) were treated with one s.c. rezafungin dose of either 5, 10, or 20 mg/kg on either day −5 (similar to day −15 for humans as noted above, day −3, or day −1. In two additional groups, mice were given either rezafungin at 5 mg/kg s.c. or amphotericin B at 3 mg/kg i.p. on day 0 administered 1 h after challenge with A. fumigatus. Mortality was observed for 14 days.

### PCP prophylaxis mouse model.

Male C3H/H3N mice (Charles River) weighing ∼0.02 kg were immunosuppressed using dexamethasone (4 mg/liter) added to drinking water acidified with sulfuric acid (1 ml/liter) to prevent secondary microbial infections. Prophylaxis was administered at the same time as inoculation with Pneumocystis murina (Cincinnati VAMC Veterinary Medical Unit, Cincinnati, OH; 2 × 10^6^/50 µl intranasally), the standard for this *Pneumocystis* infection model, given the slower growth of *Pneumocystis* relative to other fungi. Eight groups of mice (*n* = 10/group) received either negative control (control steroid, no treatment), positive control (SXT at 50/250 mg/kg 3×/week i.p.), or rezafungin (0.2, 2, or 20 mg/kg i.p. 1× or 3×/week) for 6 weeks. Mice were sacrificed after 6 weeks, and the lungs were prepared for fungal count measurement (CFU/g of tissue) of both trophic and asci (cyst) forms by rapid Wright-Giemsa and cresyl echt violet stains, respectively (57).

### Statistical analysis.

Statistical analyses were conducted according to the respective study protocols as follows. In the study of invasive candidiasis, CFU counts for each mouse were log transformed, and *P* values were calculated in Microsoft Excel by using a two-sample Student *t* test assuming unequal variance with comparisons made between treatment groups and the 24-h vehicle control group. In the study of invasive aspergillosis, a Fisher exact test (two tailed) was conducted on the survival curves. In the study of *Pneumocystis* prophylaxis, nuclei and asci counts for each lung were log transformed and analyzed by analysis of variance. Individual groups were compared by using Dunn’s test for multiple comparisons (GraphPad Prism, v6).
